# MicroRNA expression profiles during cotton (*Gossypium hirsutum L*) fiber early development

**DOI:** 10.1038/srep44454

**Published:** 2017-03-22

**Authors:** Min Wang, Runrun Sun, Chao Li, Qinglian Wang, Baohong Zhang

**Affiliations:** 1 Henan Collaborative Innovation Center of Modern Biological Breeding, Henan Institute of Science and Technology, Xinxiang, 453003, P. R. China; 2 Beijing Key Laboratory of Plant Resources Research and Development, Beijing Technology and Business University, Beijing, 100048, P. R. China; 3 Department of Biology, East Carolina University, Greenville, NC 27858, USA

## Abstract

The role of microRNAs (miRNAs) during cotton fiber development remains unclear. Here, a total of 54 miRNAs belonging to 39 families were selected to characterize miRNA regulatory mechanism in eight different fiber development stages in upland cotton cv BM-1. Among 54 miRNAs, 18 miRNAs were involved in cotton fiber initiation and eight miRNAs were related to fiber elongation and secondary wall biosynthesis. Additionally, 3,576 protein-coding genes were candidate target genes of these miRNAs, which are potentially involved in cotton fiber development. We also investigated the regulatory network of miRNAs and corresponding targets in fiber initiation and elongation, and secondary wall formation. Our Gene Ontology-based term classification and KEGG-based pathway enrichment analyses showed that the miRNA targets covered 220 biological processes, 67 molecular functions, 45 cellular components, and 10 KEGG pathways. Three of ten KEGG pathways were involved in lignan synthesis, cell elongation, and fatty acid biosynthesis, all of which have important roles in fiber development. Overall, our study shows the potential regulatory roles of miRNAs in cotton fiber development and the importance of miRNAs in regulating different cell types. This is helpful to design miRNA-based biotechnology for improving fiber quality and yield.

MicroRNAs (miRNAs) are an extensive class of endogenous, small, non-coding regulatory RNAs, which are a key factor in the posttranscriptional regulation of gene expression in almost all eukaryotes^1^,^2^,^3^,^4^,^5^,^6^. Mature miRNAs are generated from a chain of reactions that involve many enzymes^7^. General speaking, miRNAs are transcribed by RNA polymerase II into the longer self-complementary primary transcripts (pri-miRNA)^8^,^9^, then, pri-miRNAs are cleaved by RNase III-like enzyme, known as Dicer-like protein to produce miRNA precursors (pre-miRNAs)^7^,^10^. The pre-miRNA is further cleaved to a miRNA duplex (miRNA: miRNA^*^)^3^. Finally, the mature miRNA combines with RNA-induced silencing complex (RISC) to inhibit or degrade target mRNAs further to depress gene expression^11^,^12^. In plants, miRNAs targets usually have perfect or near-perfect complementary sites at the 3′ untranslated regions (UTRs) although miRNAs may also target the CDS and 5′ UTR region. Generally speaking, in plants, miRNAs perfectly bind corresponding mRNA to induce mRNA degradation^13^.

Although miRNAs are small, they play versatile roles in plant development and growth^6^. miRNAs have been implicated in the control of organ (such as leaf, stem, root and flower) development^14^,^15^, meristem cell identification^16^,^17^,^18^, phage change from vegetative growth to reproductive growth transition^19^, response to biotic and abiotic stress (salinity, drought and pathogens)^20^,^21^,^22^,^23^,^24^,^25^, and signal transduction in plants^16^,^26^. Although many plant miRNAs have been characterized, the functions of many other miRNAs still need to be validated.

Cotton fiber is a seed trichome that may grow approximately 2–3 cm after being fertilized and is thus considered the longest single cell in higher plants. Single cotton fiber cells were used to study rapid cellular elongation and cellulose synthesis^27^,^28^. The development of cotton fiber cells consists of four overlapping stages including initiation, elongation, secondary cell wall synthesis and maturation^29^,^30^. Among them, lint fiber initiates on the day of anthesis (DPA) and ends approximately at 5 DPA, which is a determining stage for the number of fibers that contribute to the cotton fiber yield^31^. By 10 DPA, single-celled fibers enter a rapid elongation stage, followed by secondary wall deposition (approximately 20 days) and maturation (after 30 days or later)^32^.

Over the past decade, a number of studies have reported that several transcription factors, such as MYB, TCP, WEKY, AP2/EREBP and bHLH, play an important role in the process of fiber initiation^33^. It has been reported that *GL1* gene is a key transcription factor regulating trichome development in *Arabidopsis*. GaMYB2, as the orthologous gene of GL1, has specifically higher expression in the early fiber initiation. Transferring the GaMYB into GL1 mutant *Arabidopsis* induced sporadic seed trichomes, suggesting that cotton fiber and *Arabidopsis* trichome may share certain regulators and GaMYB might be important to fiber trichome initiation. Fiber elongation is a complex physiological process which is regulated by several important proteins, such as vacuolar invertase, sucrose/K^+^ transporters, sucrose synthase, calcium dependent protein kinase and kinesin-like calmodulin-binding protein^34^,^35^,^36^,^37^,^38^.

At the molecular levels, fiber development is regulated by transcriptional, post-transcriptional, and translational mechanisms that impact the expression of critical protein-coding genes^33^,^39^. Although the molecular mechanism controlling cotton fiber development is not fully characterized, increasing evidence suggests that miRNAs may play an important role in the process of fiber development^40^. Indeed, several miRNA families, including miR156/157, 160, 165/166, 167, 168, 171, 396, 7505 and n22, were differentially expressed during fiber early development^40^,^41^,^42^,^43^,^44^,^45^,^46^,^47^,^48^,^49^. However, no systematic studies have been performed on miRNA expression profiling and their regulatory gene network during 4 different stages of cotton fiber development.

In this study, the expression of 54 miRNAs was investigated at eight different stages of fiber development by using qRT-PCR. To further understand the various roles of miRNAs and their corresponding regulatory networks, we predicted the potential miRNA target candidates based on the *Gossypium* (cotton) DFCI Gene Index (CGI) sequence library and approximately 3000 EST sequences. Our results suggest that the network of miRNAs and corresponding targets contributes to fiber initiation, elongation and secondary wall biosynthesis, respectively. Moreover, the result of Gene Ontology (GO) and KEGG pathway enrichment was analyzed using the miRNA targets. Interestingly, we found that miRNA targets were involved in fiber development through different metabolic pathways. Thus, our study provides better understanding of miRNA roles in cotton fiber initiation and early development.

## Results

### MiRNA expression pattern in immature ovules and fiber-bearing ovules

Cotton ovule development relies on the complex regulation of many genes. MiRNAs, small but important molecules, play a crucial role in the process of ovule development. To characterize the miRNA regulatory mechanism between immature ovule and fiber-bearing ovule, qRT-PCR was performed to analyze the expression of 54 miRNAs in ovules or fibers at eight different developmental stages.

**Figure 1:**
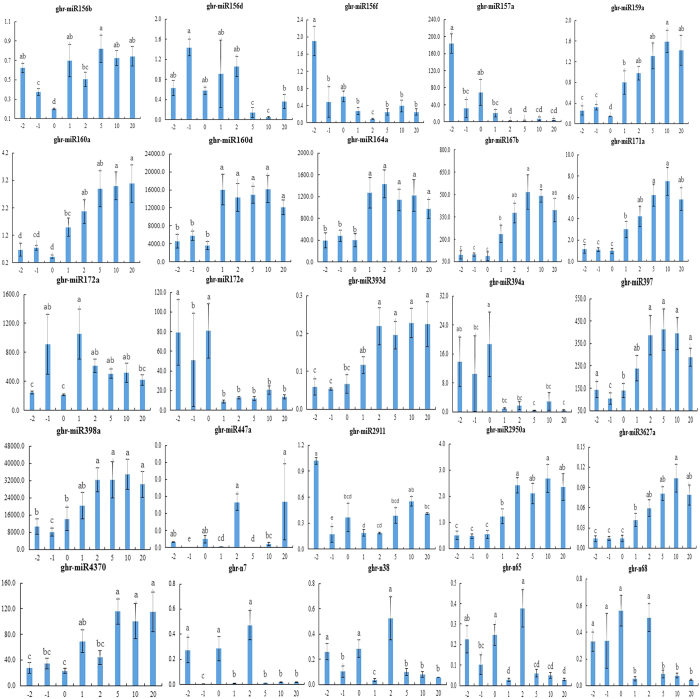
The expression of miRNAs was significantly changed as the developmental stage of cotton fiber initiation and elongation. Based on the qRT-PCR expression analysis, certain miRNAs were expressed at a higher level during initiation stage than that during elongation stage, certain miRNAs were expressed at a different way. The letter with “a, b, c, d and e” was sign via multiple comparison, which indicates significant statistical difference at *p-value* ≤ 0.05 by One-way ANOVA.

First, we focus on the miRNAs expression variation pre- and post-fiber initiation by comparing miRNA levels in ovules between −2 DPA, and 0 DPA, and then between 0 DPA and 2 DPA. Among all the miRNAs, fourteen miRNAs, including miR156b, 159a, 160a, 160d, 164a, 167b, 171a, 172a, 393d, 2950, 3627a, 397, 398a and 447a, were higher expressed in 2 DPA ovules than that in 0 DPA ovules. However, four miRNAs (miR156f, 157a, 172e and 394a) exhibited significantly lower expression in 2 DPA fiber-bearing ovules than that in 0 DPA immature ovules. Two miRNAs, miR156b and miR2911, were significantly high expressed in −2 DPA ovules than that on 0 DPA ovules (Fig. 1). Interestingly, the miR169 family, miR169a/b/c/d/h, showed a higher expression in immature ovule but had a lower expression in fiber-bearing ovule, which demonstrated that miR169 might affect immature ovule and fiber-bearing ovule via opposing mechanisms. It is worthy to consider that miRNAs coming from one family may have different expression profiles between developmental stages such as fiber-bearing ovules (miR156b, d and f) and immature ovules (172a and e) (Fig. 1).

### MiRNA expression pattern in fiber elongation and secondary wall formation

**Figure 2:**
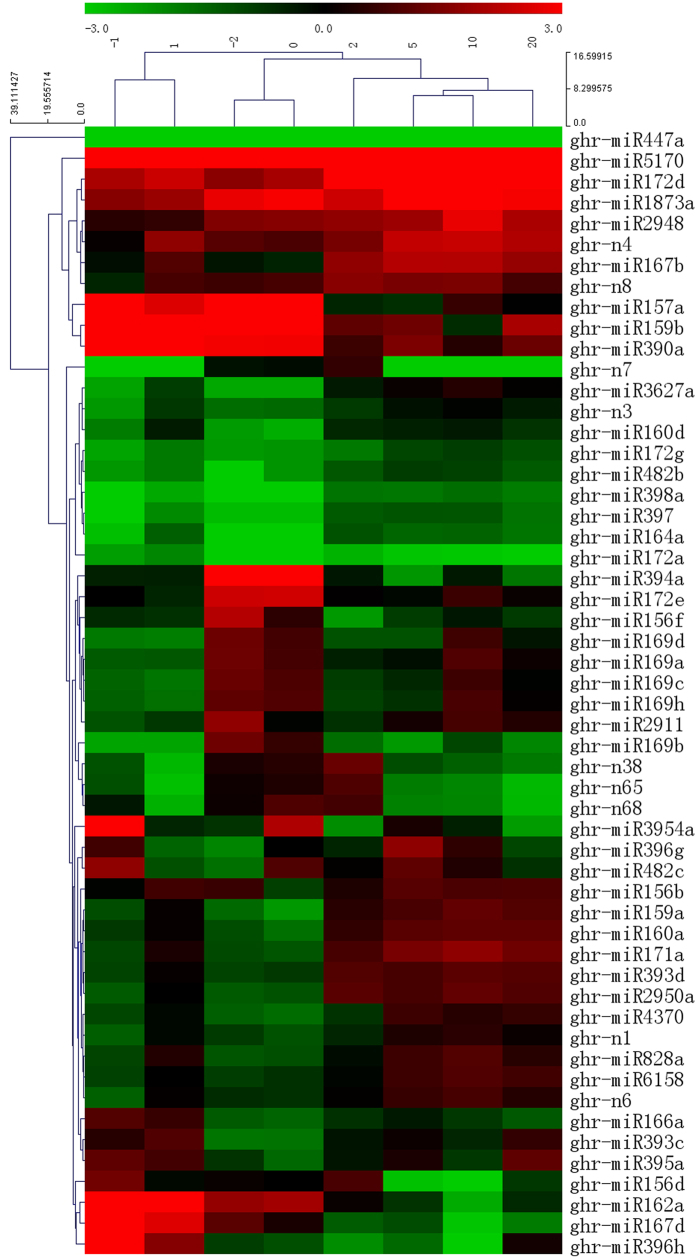
Heatmap of the expression pattern of 54 miRNAs across 8 different fiber development stages. This figure show the different expression pattern for different miRNAs. Certain miRNAs were expressed at a higher level at early stage but certain miRNAs were not. Hierarchical clustering was performed with Euclidean distance based on single linkage for each of the miRNAs among all developmental stages. In order to elucidate the regulatory role of each miRNA in different fiber development stages, a heat map was constructed by MeV4 (Multi Experiment Viewer) using Row Z-Score [(△△Ct –means)/SD]^79^,^80^,^81^. In the figures, color red, green, and black represent up-regulation, down-regulation and no change, respectively. Heat maps and clustering were done using Mev software.

The expression pattern of miRNAs in fiber elongation and secondary wall formation was also shown in Figs 1 and 2. Overall, six miRNAs (out of 54 or approximately 11%), 156d, 447a, n7, n38, n65 and n68, had a lower expression than other miRNAs while two miRNAs (miR156b and miR4370) showed higher expression in 5 DPA (fiber elongation initiation) ovules than that in 2 DPA ovules. Only miR4370 was significantly upregulated in rapid fiber elongation (10 DPA) compared with fiber elongation initiation (5 DPA). In addition, both miR156d and miR447a were upregulated in secondary wall biosynthesis compared with rapid fiber elongation. Interestingly, miR447a was the only one that was significantly expressed in the fibers of 5, 10 and 20 DPA, implying that miR447a plays crucial role in fiber elongation, fiber rapid elongation and secondary wall biosynthesis.

### A potential function of miRNA in fiber development

To further validate the potential regulatory roles of miRNAs, we constructed the miRNA expression profiles using Mev4 software (Fig. 2). The 54 tested miRNAs exhibited different expression profiles in different tissues and clustered into five major groups, each group with similar profiles. Compared with other miRNAs, nine miRNAs, including miR447a, n3, 160d, 172 g, 482b, 398a, 397, 164a and 172a, showed lower expression in all the tested tissues at all developmental stages. In contrast, the expression level of eight miRNAs (approximately 14.8%) was higher in all ovule and fiber developmental stages than that in other tissues (Fig. 2). In addition, the accumulation of three miRNAs (miR162a, 167d and 396 h) showed higher expression in immature ovules and fiber-bearing ovules than that in the fiber elongation stage. Interestingly, the expression of 12 miRNAs, miR156f, 169a/b/c/d/h, 172e, 394a, 2911, n38, n65, n68, were peaked at −2 and 0 DPA during the tested fiber development stages. The last group contained 11 miRNA members, including miR156b, 159a, 160a, 171a, 393d, 828a, 2950a, 4370, 6158, n1, and n6. This group of miRNAs was expressed at a higher level at 1, 2, 5, 10, and 20 DPA (Fig. 2). This suggests that these miRNAs may play different roles during cotton fiber initiation and early development.

### miRNA target identification and GO-based classification

**Figure 3:**
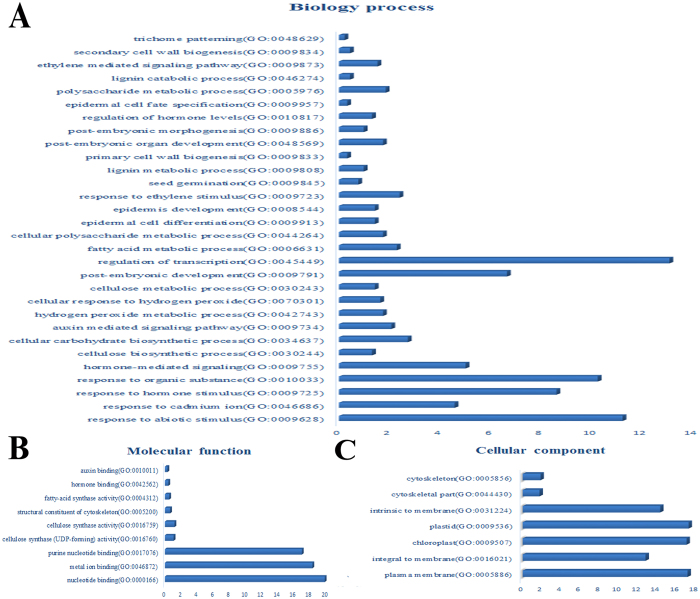
Gene ontology-based term classification of miRNA targets in cotton. (**A**) Biological process, in which more miRNAs are involved in regulation of transcription and response to different abiotic stress, (**B**) molecular function, in which more miRNAs are involved in metal ion binding and nucleotide binding, (**C**) cellular component, in which miRNA get involved in many cellular component, including chloroplast and integral to membrane.

The *Gossypium* (cotton) DFCI Gene Index (CGI) sequence library which is found in psRNATargets database^50^ was used as data source for predicting miRNA targets. This database contains all the published and unpublished cotton mRNA sequences. In total, 3567 sequences were predicted to be miRNA targets. These sequences were also used to perform homology searches against the published Refseq RNA data of *Arabidopsis* to get the homologous genes for miRNA targets. Then, the *Arabidopsis* genes that are homologous to miRNA targets were used as an input for DAVID database to investigate the potential function of cotton miRNA targets. In total, 220 biological processes, 67 molecular functions, and 45 cellular component were categorized in GO classification analysis. The top three biological processes were: regulation of transcription (GO:0045449), response to abiotic stimulus (GO:0009628), and response to organic substance (GO:0010033). miRNAs were also involved in several biological processes that are related to cotton seed and fiber development, includind trichome patterning (GO:0048629), secondary cell wall biogenesis (GO:0009834), regulation of hormone levels (GO:0010817), seed germination (GO:0009845), and cellulose biosynthetic process (GO:0030244) (Fig. 3A). Similarly, several molecular processes, including auxin binding (GO:0010011), hormone binding (GO:0042562), fatty-acid synthase activity (GO:0004312), and cellulose synthase activity (GO:0016759) are related to fiber development (Fig. 3B). It has also been reported that the cytoskeleton (GO:0005856) plays an important role in fiber development and it was the only predicted cellular component that relates to cotton fiber development (Fig. 3C).

**Figure 4:**
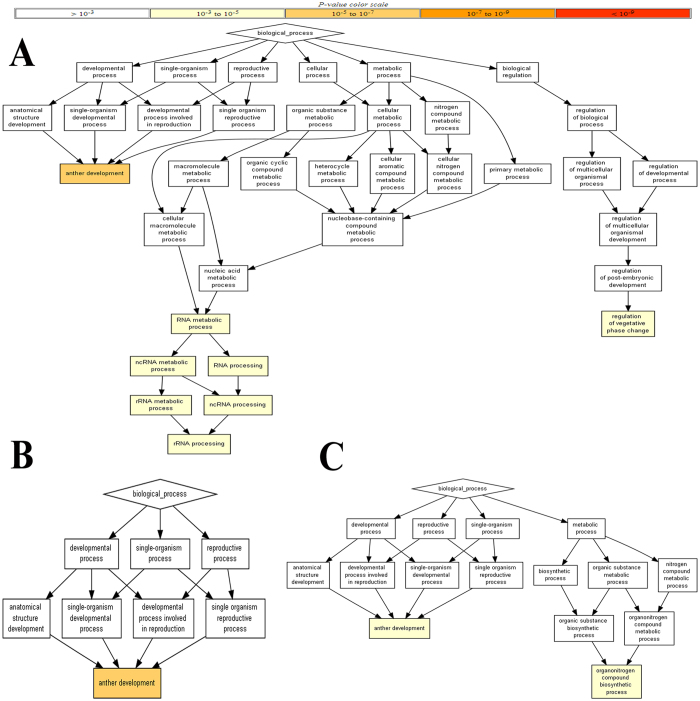
Based on Gorilla, more complicated enriched biological processes was built and miRNAs are involved in many different biological processes. (**A**) Biological process in all tested fiber stages, (**B**) biological process in fiber initiation, (**C**) biological process in fiber elongation and secondary wall biosynthesis. Yellow color represents *p-value* from 10^−5^ to 10^−3^.

To further understand the biological process of identified miRNA targets, Gorilla (process ontology) was employed to visualize the miRNA-target biological enrichment based on the relationships among enriched processes via directed acyclic graph. In which, there are six major hubs relating to plant development, reproduction, single organism, cellular and metabolic processes, and biological regulation. Three hubs are related to anther development, rRNA processing and the regulation of vegetative phase change which were also significantly enriched (Fig. 4A). In addition, we performed separate enrichment analyses for fiber initiation, elongation, and second wall formation (Fig. 4B,C). Anther development was enriched for fiber initiation, elongation and secondary wall biosynthesis. Interestingly, organo-nitrogen compound biosynthetic process was different in fiber initiation, fiber elongation and secondary wall formation, indicating that organo-nitrogen compound biosynthetic process was important to fiber elongation and secondary wall formation (Fig. 4C).

### KEGG-based pathway enrichment

**Figure 5:**
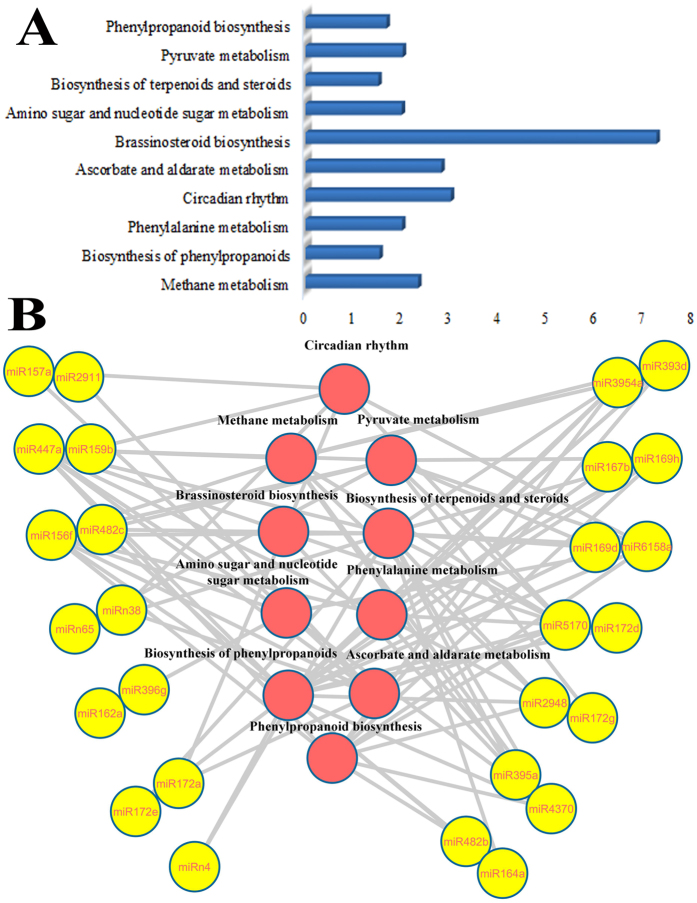
KEGG-based pathways enrichment of miRNA targets. miRNA are involved in many metabolism. (**A**) KEGG pathways of miRNA targets, (**B**) The interaction network of miRNA and corresponding enrichment pathway.

To gain further insights into the metabolisms, DAVID and KEGG database (http://www.genome.jp/kegg/) were employed to perfom KEGG-based pathway enrichment for identified cotton miRNA targets. Ten KEGG pathways, including methane metabolism, biosynthesis of phenylpropanoids, phenylalanine metabolism, circadian rhythm, ascorbate and aldarate metabolism, brassinosteroid biosynthesis, amino sugar and nucleotide sugar metabolism, biosynthesis of terpenoids and steroids, pyruvate metabolism, phenylpropanoid biosynthesis, were identified and the network between miRNAs and pathways was constructed using cytoscape (Fig. 5A,B). Of the 54 miRNAs, about half miRNAs showed KEGG enrichment (Fig. 5A). Three KEGG pathways: biosynthesis of phenylpropanoids (ath01061), circadian rhythm (ath04712) and pyruvate metabolism (ath00620), are known to be involved in lignans synthesis, cell elongation and fatty acid biosynthesis, respectively, which may play a role in fiber development.

### MiRNA target- mediated network in fiber development

**Figure 6:**
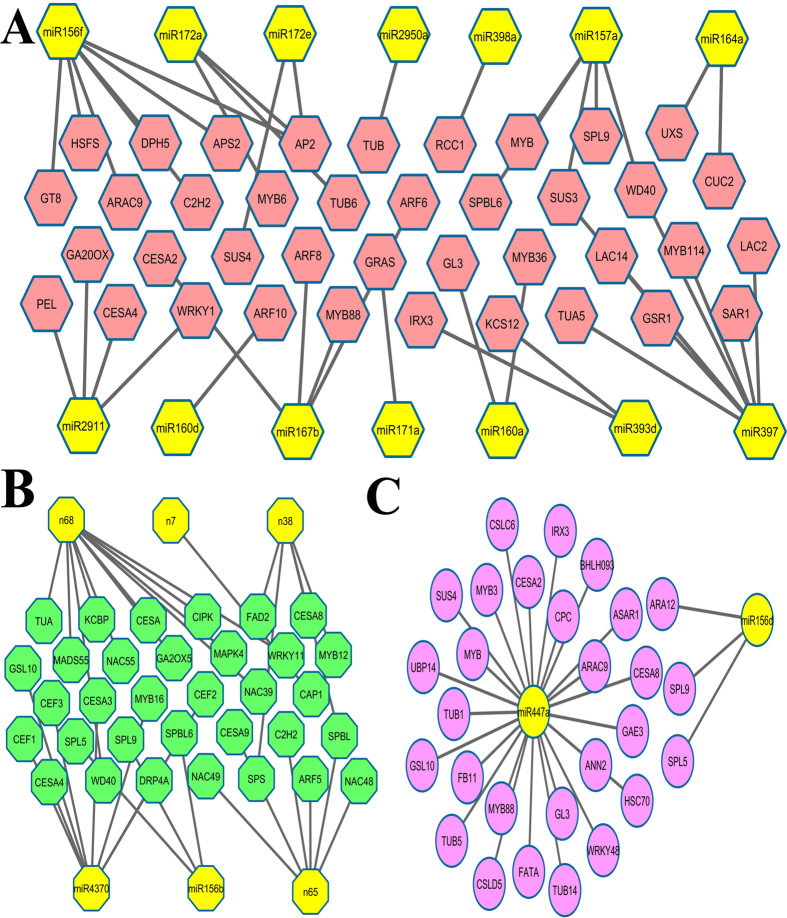
The miRNA-mediated interaction network during cotton fiber development. miRNAs regulate cotton fiber initiation and development through a complicated gene network. In this miRNA-gen network, miRNA may target a couple of protein-coding gene and involved in a multiple developmental stage. (**A**) Fiber initiation, (**B**) Fiber elongation, (**C**) Fiber secondary wall biosynthesis.

*Arabidopsis* leaf trichomes could serve as an ideal model for elucidating fiber development because the genetic mechanism of cotton fiber development shows many similar patterns to leaf trichomes of *Arabidopsis*^51^. Therefore, we searched for functional genes which play important roles in trichomes and fiber development in *Arabidopsis* and cotton, respectively. The miRNA-mediated interaction network during fiber initiation, elongation and secondary wall biosynthesis were built using Cytoscape software. As shown in Fig. 6A–C, 14 miRNAs and 43 corresponding targets were considered to be important in fiber initiation. Six miRNAs (miR156b, 4370, n7, n38, n65 and n68) and 34 corresponding targets were involved in fiber elongation. Furthermore, two miRNAs, miR156d and miR447a, play a crucial role in secondary wall biosynthesis.

## Discussion

### miRNAs are involved in cotton fiber initiation

**Figure 7:**
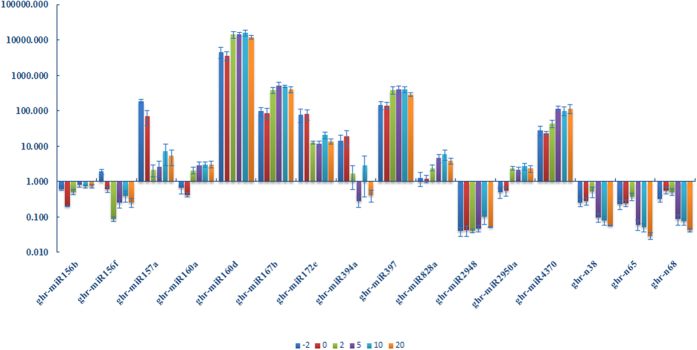
The miRNA expression in −2, 0, 2, 5, 10 and 20 DPA. Sixteen miRNAs were selected based on GO and KEGG analysis, which were aberrantly expressed during fiber initiation and development. The expression data was generated from qRT-PCR and the same as in the Fig. 2.

Certain studies have suggested that plant hormones are critical regulators in fiber development. For example, auxin is a positive regulator that promotes ovule and fiber development. It is worth noting that the accumulation of miR160d was higher in fiber-bearing ovules and fibers than that in immature ovules (Figs 2 and 7). This suggests that the putative targets that encode ARF10 had higher expression in fiber initiation which is consistent with the positive role of auxin response factors in cotton ovule development. Another auxin response factor, ARF8, was predicted to be targeted by miR167b^40^, and also proven in this present study (Fig. 6). The expression of miR167b was higher in all development stages particularly in the fiber elongation stage (Figs 2 and 7), which might indicate that ARF8 play a role in fiber initiation. Interestingly, ARF5 was newly identified as a target of miRNA n65 with a lower expression from 5 to 20 DPA (Figs 2, 6 and 7) suggesting that ARF5 may accumulate at higher levels in fiber elongation and secondary wall formation. Our study also showed that miR447a potentially targets heat shock 70 protein (HSC70). One study showed that HSC70 may participate in fiber development processes^52^. This suggests that miR447a may be involved in cotton fiber development through targeting HSC70.

MiR394a shows diverse expression profiles at different fiber development stages. The expression of miR394a is significantly higher in fiber initiation than that in later developmental stages. This suggests that miR394a may regulate cotton fiber initiation. Furthermore, the expression level of miR394a is higher in 10 DPA fiber than that in 5 DPA and 20 DPA fiber (Fig. 7), indicating that miR394a may be specifically involved in the rapid elongation of fiber.

Laccase may regulate copper homeostasis and lignin biosynthesis^53^,^54^ and its transcripts LAC2 and LAC14 were predicted to be the targets of miR397 (Fig. 6). The expression level of miR397 was high in the fiber at all developmental stages, particularly in the fiber elongation period (Fig. 7) which may result in the downregulation of the laccase genes. We speculate that, less laccase may reduce lignin production, causing the epidermal cell wall loosening. This reaction prompts fiber bulging and initiation.

### miRNAs are involved in cotton fiber elongation and secondary wall formation

Several studies have demonstrated that miRNAs may play a crucial role in the process of fiber elongation. It has been shown that the target of miR156, SPL9, plays an important role in floral organ trichome formation, fiber initiation and elongation. In our study, the expression level of miR156b/157a was decreased in the ovules from −2 DPA to 0 DPA, but increased at 5 DPA and remained relatively unchanged in the fibers at 10 and 20 DPA (Fig. 7). This result was consistent with previous reports^45^,^46^. This indicates that the target gene, SPL9, could play facilitate fiber initiation. Therefore, miR156b/157a might negatively regulate the fiber elongation via the mediation of SPL9 cleavage which is critical for the initiation and termination of fiber cell elongation. Previous studies reported that the SPL9 inhibited the expression of *DFP, F3H* and *ANS* genes to regulate the anthocyanin accumulation^55^. This might be important to adjust the H_2_O_2_ signaling in the final stages of fiber elongation^45^.

It is well known that miR172 together with miR156 regulate the transition of vegetative growth to reproductive growth in complementary patterns^56^,^57^,^58^,^59^. The downregulation of miR156 promotes SPL9 expression, which results in an increase of miR172 expression. In our study, we found that the expression of miR156b, generally speaking, was lower during cotton fiber initiation and then increased during cotton fiber elongation. In contrast, miR172e, showed a different pattern, in which miR172e was expressed at a higher level during fiber initiation than that in later stages (Fig. 7). This suggests that the miR156 and miR172 may co-regulate the cotton fiber initiation and development.

Cellulose synthase^60^, using uracil-diphosphate (UDP)-glucose as one of the substrate to synthesize cellulose, was enriched in elongating fiber^61^. Nine CESA homologs were reported to regulate primary wall biosynthesis and six CESAs were related to secondary wall biosynthesis in *G. raimondii* genome^62^. In this study, two CESAs (CESA8 and CESA9) were predicted to be targeted by miRn38, n65 and n68. The expression of miRn38, 65 and 68 was diverse in the tested tissues and relatively lower during fiber elongation and secondary wall biosynthesis (Figs 2 and 7). This suggests that the accumulation of CESAs was increased in fiber elongation and secondary wall biosynthesis, which is helpful to synthesis cellulose.

Disrupting the expression of sucrose synthase genes can change fiber cell development^63^. For instance, the suppression of *GhSUS* gene expression decreased the cellulose content in fiber cell walls^64^. In our study, SUS4 was predicted to be a target of miR172e whose expression was lower in 5 to 20 DPA fiber than that in other developmental stages (Figs 2, 6 and 7). We speculate that the expression of SUS4 is higher in fiber elongation and secondary wall formation, which might increase the cellulose content in fiber cell wall.

MYB transcription factors are involved in leaves and fibers trichome development in cotton^65^,^66^,^67^. Several MYBs are predicted to be targeted by certain miRNAs. MYB 36, 88, and 114, are potentially targets of miR160a, 167b, 397, respectively. MYB 12 and MYB 16 are the target of miRn38 and miR4370. MYB3 and MYB88 are targeted by miR447 which was significantly expressed in all tested tissues. It has been reported that MYB protein family members are putative targets of some poorly conserved miRNAs, such as miR399 and miR828^40^. In our study, three MYB genes: MYB 2, MYB 3 and MYB 12, were predicted to be targeted by miR828. The high expression levels of miR828a during fiber elongation suggests that miR828a plays negative role in cotton elongation (Figs 2 and 7).

miR2948 regulates the expression of a couple of genes affecting cotton ovule and fiber development. Two predicted targets of miR2948, encoding sucrose synthase and glucose-methanol-choline oxidoreductase, were previously reported. Among them, sucrose synthase regulates fiber initiation and elongation^64^. In this study, TUA2 and IAA14 were predicted to be targeted by miR2948. The expression of miR2948 was low in all fiber developmental stages, suggesting that the TUA2 and IAA14 might have a high expression (Fig. 7). Hence, miR2948 may play a potential role in fiber developmental stage. It has been demonstrated that gibberellin 3-hydroxylase 1 is a putative target of miR2950 and this gene was shown to control internode elongation in pea^68^. It seems likely that gibberellin may effect fiber cell development. However, TUB was predicted to be targeted by miR2950 in this study. TUB was shown to be highly expressed in elongating fiber cells when compared with fuzzless lintess mutant ovules^69^. Interestingly, the miR2950a was poorly expressed in immature ovules (Fig. 7). The expression of miR2950a was higher in the fiber elongation stage than that in immature and fiber-bearing ovules, indicating that TUB has lower expression in fiber elongation. This result was opposite to previous reports, suggesting that the concentration of TUB might be also influenced by other metabolic processes.

### The regulation role of miR447a

miR447a was the only miRNA that was significantly expressed during different fiber development stages including fiber initiation, elongation and secondary wall biosynthesis. The targets of miR447a include many genes and transcription factors, such as ACT7, Ann2, bHLH093, CPC, GL3, MYB16, MYB88 and TUB1. The majority of target genes have been reported to regulate trichome development in *Arabidopsis*. The abundance of miR447 targets might imply the pleiotropic functions of miR447, which is consistent with the significant expression in different fiber developmental stages.

The regulatory network of miRNAs in ovule and fiber development is very complicated. By using qRT-PCR, we examined and validated the expression profiles and patterns of 54 miRNAs which were involved in fiber differentiation and early development. We identified candidate miRNA targets that were shown to be involved in fiber development via KEGG and GO analyses. We also provided the miRNA-mediated regulation network in fiber initiation, elongation and secondary wall formation. The outcomes of the present study highlight the miRNA regulatory roles in cotton fiber development and will pave the foundation to increase cotton fiber quality and yield.

## Materials and Methods

### Plant growth and material collection

Upland cotton (*Gossypium hirsutum* L.) cv. Baimian 1 (BM-1), bred by Henan Institute of Science and Technology (HIST), was planted in the greenhouse of East Carolina University under the regular agronomic conditions. Flowers were tagged on the day of anthesis (0 DPA), and the stage of 1 and 2 days prior to anthesis was estimated by the flower bud shape and size. The cotton bolls of −2, −1, 0, 1, 2, 5, 10 and 20 DPA were harvested with 6 biological replicates. Ovules of −2, −1, 0, 1 and 2 DPA were carefully dissected from each boll, fiber of 5, 10 and 20 DPA was quickly separated from ovules using forceps. Samples were immediately frozen in liquid nitrogen and then stored in −80 °C freezer for later total RNA extraction.

### Total RNA isolation and reverse transcription

Total RNA in different ovules and fibers was extracted using the mirVana™miRNA Isolation Kit (Ambion, Austin, TX, USA) according to our previous reports^70^,^71^. Briefly, tissues were first ground in liquid nitrogen using a mortar and samples were quickly transferred into 2 ml micro-tubes with 600 μL lysis/binding buffer. 10 μL miRNA homogenate additive was added and the sample was vortexed several times to mix well. After washing three times by using washing solution 1 and 2, and 3, 60 μL 95 °C RNase-free water was added to the filter for RNA elution. The quality and quantity of isolated RNAs were measured by NanoDrop-1000 (NanoDrop Technologies, Wilmington, DE, USA).

For each reverse transcription reaction, 200 ng total RNA was used to transcribe into cDNA using TaqMan^®^ MicroRNA Reverse Transcription Kit (Applied Biosystems, Foster City, CA, USA) with a miRNA-specific stem loop primer. According to the kit protocol, reverse transcription reaction was performed as following: 30 min at 16 °C, followed by 30 min at 42 °C, then 5 min at 85 °C and finally holding at 4 °C. When the reverse transcription was done, 80 μL DNase free water was added to dilute the product and vortexed gently to mix thoroughly then kept at −20 °C for later qRT-PCR analysis.

### Quantitative real-time PCR

A total of 54 miRNAs were selected to investigate their expression profiles and changes during cotton fiber development. These 54 miRNAs were selected based on previous reports^40^,^72^,^73^, in which these miRNAs show differential expression in certain developmental stages and these miRNAs may be associated with fiber initiation and development. The expression of 54 miRNAs was detected and quantified by quantitative real-time PCR using an Applied Biosystems ViiA 7 Real Time PCR System (Foster City, CA, USA) as reported previously^70^,^74^,^75^. These reactions were performed in 384 well plates and each reaction contained 2 μL forward and reverse primers (0.2 μM), 1 μL cDNA template (from 15 μL reverse transcription products of 200 ng total RNAs and then diluted by 80 μL DNase free water), 2 μL DNase free water and 5 μL SYBR green fluorescent probe. In order to minimize the chance of error, three biological replicates with three technical replicates were run for each miRNA within each fiber developmental stage.

The Ct values of all tested tissues were exported to calculate the fold change. Several studies show that multiple genes as reference genes enables gene expression normalization, recently, more and more research used the average of multiple genes’ Ct values for normalization^76^,^77^,^78^. In this study, we adopted the same approach and the average of 54 miRNA Ct value was used to obtain ∆Ct. Then, we calculated the fold change (2^−△△Ct^) in each tested development stage. Statistical analyses were based on One-way ANOVA via SPSS.

In order to elucidate the regulatory role of each miRNA in different fiber development stages, a heat map was constructed by MeV4 (Multi Experiment Viewer) using Row Z-Score [(∆∆Ct –means)/SD]^79^,^80^,^81^. Hierarchical clustering was performed with Euclidean distance based on single linkage for each of the miRNAs among all developmental stages. Fold change values were used to construct heat maps coupled with non-suppervised hierarchical clustering using Euclidean distance and complete linkage analysis.

### Prediction of miRNA targeted genes

The web tool of *psRNATarget* (http://plantgrn.noble.org/psRNATarget/) was employed to search miRNA targets using the *Gossypium* (cotton) DFCI Gene Index (CGI) sequence library 11 with the following criteria: original defined maximum expectation and maximum energy to unpair the target site^82^, the hspize value in the range from 1 to 17 nt, no central mismatch between 10 and 11 nt. The central complementary region is essential for cleavage as previously reported because the mismatch of miRNA and targets often happens around the central region^83^, that’s why we set the mismatch in 10 and 11 site which will reduced false positive significantly.

### GO classification and KEGG pathway analysis

Gene Ontology (GO) summarizes information about the function of gene products in three different ways that includes the molecular function, biological processes and cellular components. KEGG (Kyoto Encyclopedia of Genes and Genomes) pathway enrichment is also utilized for bioinformatics research that aims to investigate the cellular and organismal functions of tested genes. To visualize the Gene Ontology classification and KEGG pathway enrichment of cotton miRNA targets, *Arabidopsis* gene homologs for the identified miRNA targets were used as input data for David database (http://david.abcc.ncifcrf.gov/). To visualize the relationships among miRNA targets, we used GOrilla (process ontology) software to construct a directed acyclic graph (http://cbl-gorilla.cs.technion.ac.il/), GOrilla provides DAG (directed acyclic graph) showing relationships among enriched processes.

## Additional Information

**How to cite this article:** Wang, M. *et al*. MicroRNA expression profiles during cotton (*Gossypium hirsutum L*) fiber early development. *Sci. Rep.* **7**, 44454; doi: 10.1038/srep44454 (2017).

**Publisher's note:** Springer Nature remains neutral with regard to jurisdictional claims in published maps and institutional affiliations.
